# Development of a Temperature-Switch PCR-Based SNP Typing Method for *Mycobacterium ulcerans*

**DOI:** 10.1371/journal.pntd.0001904

**Published:** 2012-11-15

**Authors:** Katharina Röltgen, Kobina Assan-Ampah, Emelia Danso, Dorothy Yeboah-Manu, Gerd Pluschke

**Affiliations:** 1 Swiss Tropical and Public Health Institute, Molecular Immunology, Basel, Switzerland; 2 University of Basel, Basel, Switzerland; 3 Noguchi Memorial Institute for Medical Research, University of Ghana, Legon, Ghana; University of Tennessee, United States of America

## Abstract

*Mycobacterium ulcerans* (*M. ulcerans*), the causative agent of the devastating skin disease Buruli ulcer (BU), is characterized by an extremely low level of genetic diversity. Recently, we have reported the first discrimination of closely related *M. ulcerans* variants in the BU endemic Densu River Valley of Ghana. In the study real-time PCR-based single nucleotide polymorphism (SNP) typing at 89 predefined loci revealed the presence of ten *M. ulcerans* haplotypes circulating in the BU endemic region. Here we describe the development of temperature-switch PCR (TSP) assays that allow distinguishing these haplotypes by conventional agarose gel-based analysis of the PCR products. After validation of the accuracy of typing results, the TSP assays were successfully established in a reference laboratory in Ghana. Development of the cost-effective and rapid TSP-based genetic fingerprinting method will thus allow investigating the spread of *M. ulcerans* clones by regular genetic monitoring in BU endemic countries.

## Author Summary

As the third most common mycobacterial disease after tuberculosis and leprosy, Buruli ulcer constitutes a considerable health problem in many parts of the world, but in particular in West and Central Africa. Although this emerging skin disease is commonly associated with proximity to aquatic habitats, the mode of transmission remains obscure. While the clonal population structure of *Mycobacterium ulcerans* provides a great potential to trace transmission pathways and evolutionary relationships, micro-epidemiological studies have long been hampered by a striking lack of genetic diversity among African *M. ulcerans* populations. Whole genome comparison of *M. ulcerans* strains isolated from patients living in the BU endemic Densu River Valley of Ghana led to the identification of single nucleotide polymorphisms between these closely related strains. The subsequent development of SNP typing assays enabled a differentiation of ten *M. ulcerans* haplotypes in the BU endemic region. Here we selected canonical SNP markers for a spatio-temporal analysis of *M. ulcerans* variants in the Densu River Valley of Ghana using a temperature-switch PCR-based approach.

## Introduction

Infection with *M. ulcerans* causes a chronic and necrotizing skin condition known as Buruli ulcer. This emerging disease occurs focally in more than 30 predominantly tropical countries worldwide, but mainly affects impoverished populations of West and Central Africa with limited access to health care services [1]. Recent findings suggest that *M. ulcerans* has diverged about a million years ago from the fish pathogen *Mycobacterium marinum* by the acquisition of a plasmid encoding the enzymes required for the production of mycolactone [2]–[4]. Mycolactone is a cytotoxic macrolide toxin that plays a key role in the unique pathology of BU [5], characterized by the formation of progressive skin ulcers. While a potential transmission model implicating mammals as reservoirs and mosquitoes as vectors of *M. ulcerans* has been proposed for a local BU endemic region in south-eastern Australia [6], [7], epidemiologic information for BU endemic African settings is sparse.

Remarkably little genetic diversity between *M. ulcerans* isolates from African BU patients has hindered molecular epidemiological studies tracing the spread of genetic variants of *M. ulcerans*. However, from a phylogenetic perspective the genetic monomorphism of this pathogen, which is associated with a clonal ancestry, holds a great potential to trace transmission pathways and evolutionary relationships. Several studies of other genetically highly homogeneous pathogens such as *Mycobacterium leprae* [8], *Bordetella pertussis* [9], *Yersinia pestis* [10] and *Bacillus anthracis* [11] have demonstrated the validity of genome-wide single nucleotide polymorphisms as markers for such phylogenetic analyses.

In our previous work we compared genome sequences of three Ghanaian *M. ulcerans* isolates, selected on the basis of the three earlier identified variable number of tandem repeat (VNTR) types among 57 *M. ulcerans* strains from Ghana [12], in order to detect a comprehensive set of SNP markers for genotyping studies. The subsequent development of 65 real-time PCR-based typing assays for the identified SNP loci allowed us to differentiate 75 *M. ulcerans* strains from a BU endemic area in the Densu River Valley of Ghana into six haplotypes. Genome re-sequencing of four haplotype representatives followed by further SNP detection and design of 24 additional real-time PCR assays led to the identification of ten haplotypes (HT1–10) among the 75 *M. ulcerans* isolates (Table 1).

**Table 1:** Summary of *M. ulcerans* genotyping in Ghana.

Genotyping method	No. of isolates	Origin (year of isolation)	No. of loci	No. of genotypes
[12] VNTR	57	Ghana (1997–2004)	2	3
[13] SNP^1^	75	Densu river (1999–2007)	65	6
[13] SNP^2^	75	Densu river (1999–2007)	**89**	**10**
canSNP	24	Densu river (2009–2011)	**10**	**10**

1 genotyping after comparison of 3 *M. ulcerans* genomes.

2 genotyping after comparison of 7 *M. ulcerans* genomes.

Here we report the development of a simplified and cost-effective SNP typing method based on TSP and analysis of PCR products by conventional agarose gel electrophoresis. For this approach we have selected ten canonical SNP (canSNP) markers that facilitate a rapid differentiation of the ten described *M. ulcerans* haplotypes in the Densu River Valley of Ghana by the elimination of diagnostically redundant assays. This strategy is used to monitor the temporal as well as spatial distribution and spread of the *M. ulcerans* haplotypes in that region. Results of this ongoing study are expected to provide insights into the circulation of *M. ulcerans* variants in a BU endemic area.

## Materials and Methods

### Ethics statement

*M. ulcerans* isolates analyzed in this study were cultivated for BU diagnosis. Ethical approval to use the isolates for immunological and microbiological research was obtained from the institutional review board of the Noguchi Memorial Institute for Medical Research, University of Ghana, Legon, Ghana (Federal-wide Assurance number FWA00001824). Written informed consent was provided by all patients involved in this study.

### Mycobacterial strains and genomic DNA extraction

We analyzed a total of 33 *M. ulcerans* isolates cultivated from wound specimen of BU patients living in the BU endemic Densu River Valley of Ghana. Ten of these isolates, used for the setup of SNP typing assays, had been typed previously by real-time PCR as SNP haplotypes 1–10 (Agy99 (HT1), NM98/03 (HT2), NM83/03 (HT3), NM100/03 (HT4), NM27/02 (HT5), NM18/02 (HT6), NM74/03 (HT7), NM32/02 (HT8), NM28/02 (HT9), and NM78/03 (HT10)) [13]. Genomic *M. ulcerans* DNA was isolated by cell wall disruption and phenol-chloroform extraction as described earlier [14]. DNA was quantified by using Qubit Fluorometer (dsDNA HS Assay Kit, Invitrogen).

### Selection of single nucleotide polymorphism loci and TSP assay design

**Figure 1:**
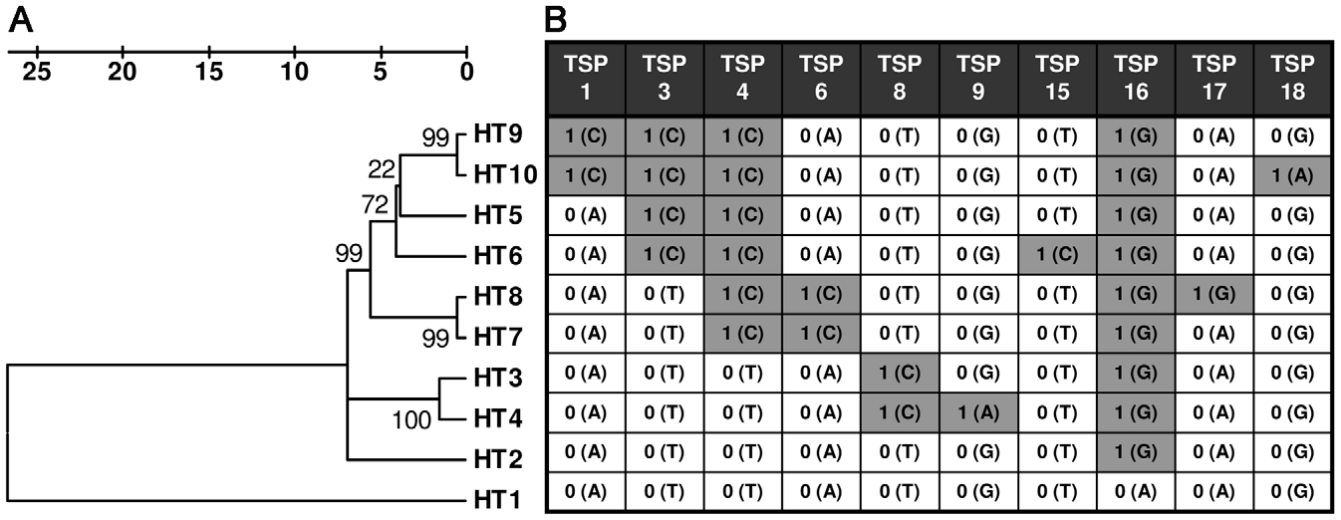
Selection of TSP SNP typing assays. **A** Linearized phylogenetic tree of the ten *M. ulcerans* haplotypes (HT1–10) detected in the Densu River Valley of Ghana (MEGA software version 4.1 (beta), scale: number of differences at the 89 SNP loci tested). **B** Schematic overview of reference (0) and SNP (1) alleles present in the sequence of the ten *M. ulcerans* haplotypes, which can be identified by TSP assays 1, 3, 4, 6, 8, 9, 15, 16, 17 and 18.

**Figure 2:**
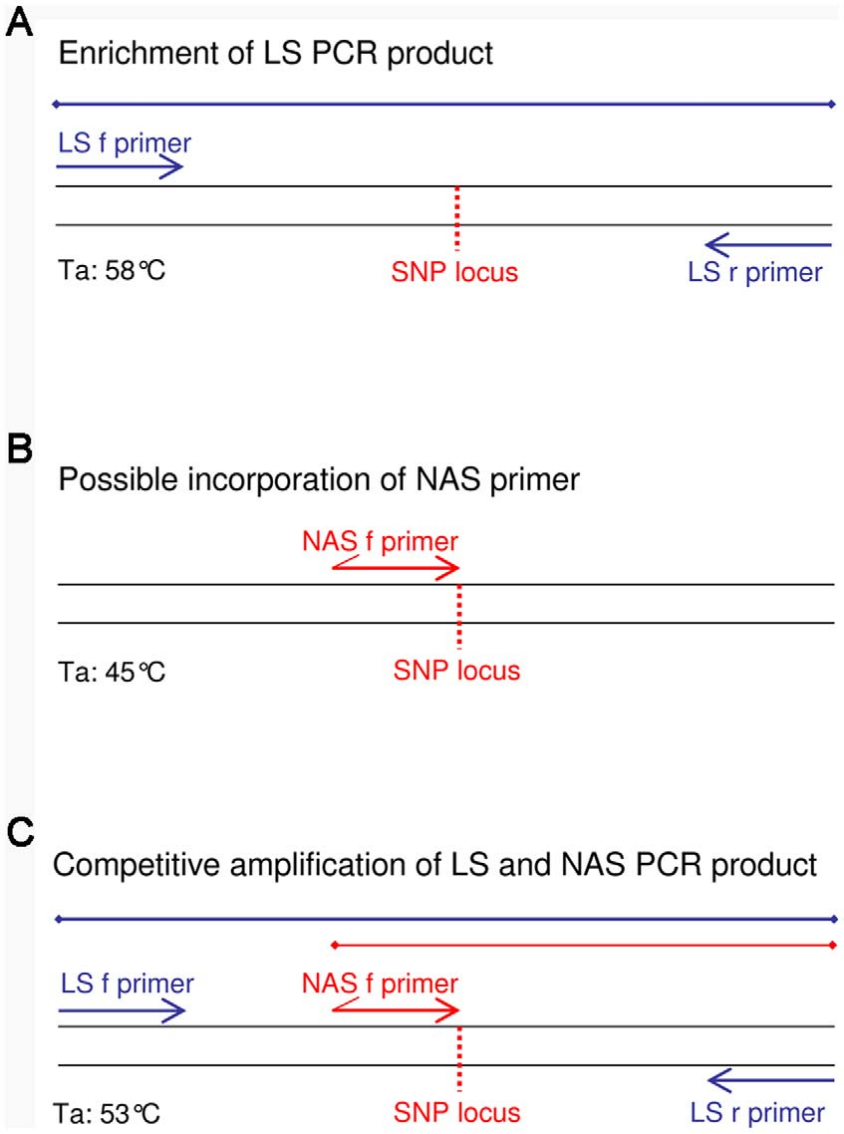
Schematic illustration of TSP assay performance. NAS and LS primer locations relative to the *M. ulcerans* DNA sequence surrounding a SNP locus are shown for the different PCR reaction phases. **A** Initial PCR conditions enable an amplification of the larger LS PCR product by applying an annealing temperature (Ta) of 58°C. **B** Reduction of Ta to 45°C facilitates a possible incorporation of the NAS primer into the enriched LS PCR product. **C** Competitive amplification of the larger LS and the smaller NAS PCR products are ensured by an increase of Ta to 53°C.

In our previous work we have detected ten *M. ulcerans* haplotypes (HT1–10) in a relatively small BU endemic region within the Densu River Valley of Ghana by amplification refractory mutation system (ARMS) real-time PCR assays [13]. Here we constructed a phylogenetic tree of the ten identified haplotypes based on concatenated sequences of the 89 SNP loci (Figure 1A). For the TSP assay development, we selected ten representative SNP loci (TSP1, 3, 4, 6, 8, 9, 15, 16, 17 and 18), providing a discrimination of all described *M. ulcerans* variants in the Densu River Valley by haplotype-specific allele combinations (Figure 1B). TSP assay primers were designed on the basis of the strategy described by Hayden and Tabone [15], [16]. For each SNP marker, locus specific (LS) primers amplifying the region surrounding the SNP of interest as well as a nested allele-specific (NAS) primer fully complementary to the sequence of reference strain Agy99, but mismatched at the 3′end nucleotide for *M. ulcerans* strains harboring the SNP at this locus, were designed using Primer 3 Software [17] (Figure 2, Table S1). LS primers were designed to have an optimum melting temperature (Tm) of 63°C (range of 62–64°C) and to amplify a PCR product greater than 400 bp. The NAS primer was designed to have a core region with an optimum Tm of 46°C (range of 43–48°C) and a non-complementary 5′tail region that increased the overall optimum primer Tm to 53°C (range of 52–55°C). The forward (TSP assays 1, 3, 4, 6, 8, 9, 15, 16) or reverse (TSP assays 17, 18) NAS primer was positioned in at least 60 bp distance from the corresponding forward/reverse LS primer to ensure a clear distinction between the larger LS and the smaller NAS PCR product. Primer sequences and PCR product sizes are provided in Table S1.

### Differentiation of alleles by TSP and analysis of PCR product size

The TSP method described by Hayden and Tabone [15], [16] served as basis for the development of a TSP assay protocol for *M. ulcerans*. A number of technical details were modified, including standard PCR reagents, the addition of diluted DNA instead of DNA desiccation and the usage of a three-primer system with optimized primer concentrations. PCR assays were performed using 0.5 U FIREPol DNA Polymerase (Solis BioDyne), Buffer BD, 3 mM MgCl_2_, 0.25 mM dNTPs (Sigma), 0.1 µM each of forward and reverse LS primer, 1 µM (0.25 µM, 0.5 µM, 0.75 µM) of forward/reverse NAS primer and 1–50 ng genomic DNA in a total reaction volume of 5 µl. In order to avoid evaporation of the relatively small reaction volume, PCRs were carried out in 0.2 ml eppendorf PCR tube strips, which could individually be closed after addition of the reagents. PCRs were performed in a T-Professional thermocycler (Biometra).

Thermal conditions for PCR amplification of *M. ulcerans* genomic DNA after an initial denaturation step (95°C for 5 min) were as follows: 15 cycles of 95°C for 30 s, 58°C for 30 s, 72°C for 1 min in order to enrich the LS product at a relatively high annealing temperature (Figure 2A); 5 cycles of 95°C for 10 s and 45°C for 30 s to enable a possible incorporation of the NAS primer into the enriched LS PCR product at a low annealing temperature (Figure 2B); 15 cycles of 95°C for 10 s, 53°C for 30 s and 72°C for 5 s to facilitate a competitive amplification of LS and NAS PCR products (Figure 2C); final extension step of 72°C for 10 min. 1 µl of the PCR products were analyzed on 2% agarose gels and stained for 1–2 hours in an ethidium bromide bath (1 µg/ml in 0.5xTBE). Whether the tested *M. ulcerans* strains harbored reference or SNP allele at the analyzed loci could then be determined by the presence of either the smaller or the larger PCR product, respectively.

### TSP validation by real-time PCR SNP typing

All TSP SNP typing results were validated by the recently described amplification refractory mutation system real-time PCR SNP typing technique [13].

## Results

### Optimization of TSP assays

TSP parameters described by Tabone et al. included the desiccation of genomic DNA by evaporation prior to PCR amplification [16]. In order to reduce the risk of contamination of the laboratory with DNA template, we eliminated this step. The addition of *M. ulcerans* DNA dissolved in water necessitated a new setup for all assay parameters.

**Figure 3:**
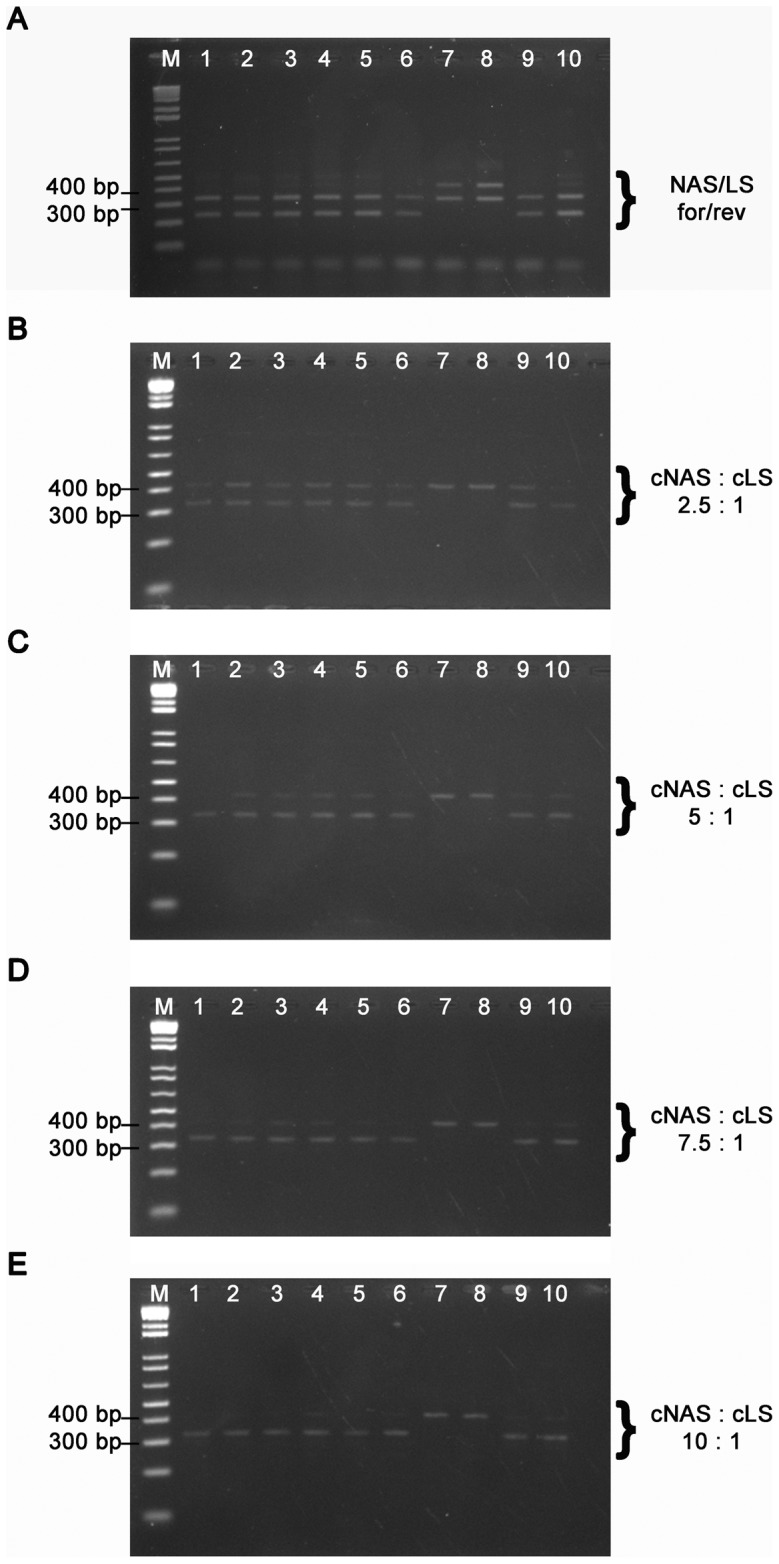
Optimization of TSP SNP typing assays. TSP endpoint detection by analysis of PCR product sizes on ethidium bromide-stained agarose gels for haplotypes 1–10 (lanes 1–10) at TSP assay locus 6. TSPs were performed using: **A** a four-primer system including both NAS and LS forward and reverse primers as well as a three-primer system with only one NAS primer and different NAS∶LS primer ratios of **B** 2.5∶1, **C** 5∶1, **D** 7.5∶1 and **E** 10∶1.

While standard PCR reagents were used according to the manufacturer's (Solis Biodyne) recommendations, the most critical step for a specific detection of either the smaller NAS or the larger LS PCR product was to identify the optimal application of NAS and LS primers used for the PCR assays. Since initial PCR reactions using a four-primer system including both forward and reverse NAS and LS primers led to the amplification of additional non-specific PCR products (Figure 3A), we applied a three-primer system with only one NAS primer. In order to ensure an accurate differentiation of reference and SNP alleles we determined optimal primer concentrations by performing TSP assays for the ten haplotype representatives at NAS∶LS primer ratios of 2.5∶1, 5∶1, 7.5∶1 and 10∶1. Results of all TSP assays provided a correct differentiation of reference and SNP alleles for the four NAS primer concentrations (Figure 3B–E). However, a clear-cut visualization of only the allele-specific PCR product was obtained by deploying a tenfold concentration of the NAS primer compared to the LS primers (Figure 3E).

The illustration of allele-specific PCR products could be further improved by loading only one-fifth of the PCR reaction on a 2% agarose gel. Gels initially overloaded by the whole PCR reaction volume showed traces of the non allele-specific PCR products.

The optimal amount of DNA for the TSP assay reactions was assessed by performing TSP assays for the ten haplotype representatives with a NAS∶LS-primer ratio of 10∶1 and different genomic DNA concentrations ranging from 1–50 ng. TSP assays provided accurate results for all DNA concentrations tested. However, subsequent standardized TSP strain typing was performed using 5 ng DNA for each reaction. The performance of TSP assays was dependent on the purity of the extracted DNA. While samples with a high ratio of OD 260/280 (>1.7) facilitated a clear visualization of allele-specific PCR products on the agarose gels, traces of non-allele-specific PCR products were detected when using DNA extracts with poorer quality. TSP assays were evaluated on ten haplotype representatives in order to confirm accurate performance of all assays for both reference and SNP alleles (Figure S1).

### TSP typing of clinical *M. ulcerans* isolates

We typed a total of 23 *M. ulcerans* strains isolated between 2009 and 2011 from BU patients living in the Densu River Valley of Ghana by the ten developed TSP assays using the optimized single standard condition (Figure S2). One additional strain served as a control for either the NAS or the LS PCR product amplification. Based on the resulting allele combinations at tested SNP loci, isolates could be differentiated into seven of the ten described *M. ulcerans* haplotypes (HT1 (1 strain), HT2 (7 strains), HT3 (1 strain), HT5 (3 strains), HT6 (5 strains), HT7 (1 strain) and HT9 (5 strains). ARMS real-time PCR SNP typing of the 23 isolates at the ten SNP loci confirmed the accuracy of TSP SNP typing results. In addition to clinical *M. ulcerans* strain typing, TSP assays were performed using DNAs directly extracted from BU lesion specimens. We selected four samples out of a panel of DNA extracts with the highest *M. ulcerans* DNA concentrations detected by IS*2404* real-time PCR. However, all attempts to SNP-type these samples by TSP failed, since additional, unspecific PCR products were amplified.

### Establishment of TSP assays in a BU endemic country

**Figure 4:**
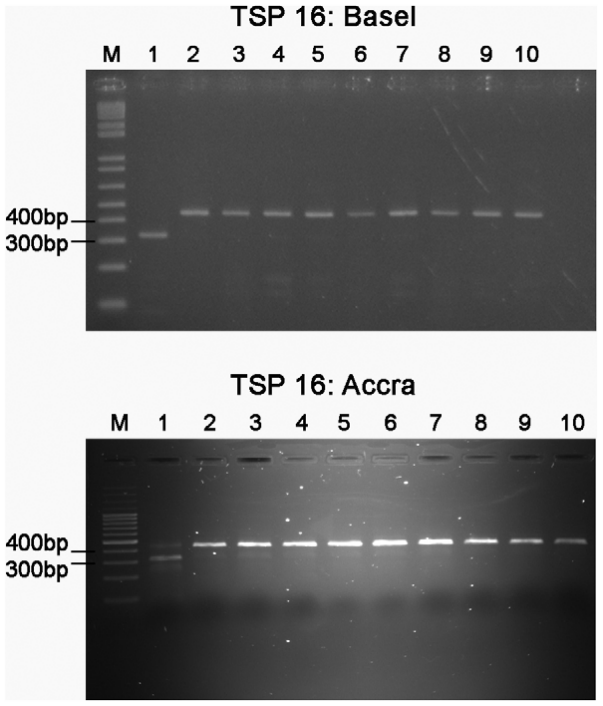
Application of TSP assay 16 in two different research laboratories. Comparison of TSP endpoint detection by analysis of PCR product sizes on ethidium bromide-stained agarose gels in two different laboratories in Basel, Switzerland and Accra, Ghana. PCR products are shown for haplotypes 1–10 (lanes 1–10).

Robustness of the assay protocol and suitability of the technique for technology transfer to laboratories in BU endemic countries was verified by analyzing TSP assays in a laboratory of the Noguchi Memorial Institute in Accra, Ghana. For this purpose we tested the ten haplotype representatives again using the same PCR materials and optimized assay parameters. Each assay provided the expected TSP genotyping products and endpoint detection by 2% agarose gels and ethidium bromide staining was comparable to the TSP setup results (Figure 4).

## Discussion

A global overview of genetic diversity in *M. ulcerans* could be established by comparative genomic hybridization analysis detecting insertional-deletional variation in 35 clinical *M. ulcerans* isolates of world-wide origin [18], [19]. However, efforts made to resolve the population structure of *M. ulcerans* strains from the same BU endemic countries were limited by the extensive genetic monomorphism of closely related isolates [12], [20]–[25]. Genome sequencing of several clinical *M. ulcerans* isolates from a BU endemic region in the Densu River Valley of Ghana has facilitated for the first time the development of a SNP-based genotyping strategy with sufficient resolution for micro-epidemiological studies. This real-time PCR-based approach provided first insights into the actual diversity of such closely related *M. ulcerans* isolates as well as the distribution of *M. ulcerans* variants in the BU endemic area. Among 75 *M. ulcerans* strains isolated between 1999 and 2007 ten haplotypes showing different patterns of geographical distribution could be detected. The identification of geographically clustered emerging haplotypes provides a valuable opportunity to monitor the spatio-temporal spread of haplotypes in this region.

Cost is an important issue for all genetic fingerprinting analyses, and this particularly applies to neglected pathogens like *M. ulcerans*. While the established real-time PCR technique required relatively expensive equipment, the TSP-based assays described here, provide a simple and cheap alternative system, thus increasing the access of genetic typing assays for *M. ulcerans* to laboratories in tropical BU endemic areas. Low cost for the PCR assays, direct endpoint detection through PCR product staining on agarose gels and simple implementation of the method provided a practical means for rapid and cost-effective SNP typing.

The critical step for TSP assay development was to design primers with optimal melting temperatures for the envisaged reaction phase participations. Efficient primer design enabled the performance of all TSP assays under a single optimized standard condition. Genotyping accuracy and robustness of the TSP strategy was demonstrated by the successful application of the designed assays in two different research laboratories, yielding identical results.

SNPs represent highly stable phylogenetic markers in genetically homogenous pathogens as they occur at very low rates and convergent or reverse mutations are highly unlikely [26]. Numerous genotyping studies of monomorphic pathogens have demonstrated that a limited set of SNPs can be used to define phylogenetic relationships [11],[27]–[30]. The idea of a canonical SNP, a SNP that can be used to define species [31], major genetic lineages of a species [27], [28] or even specific strains [32], [33] has recently been described in a review article focussing on the molecular epidemiology of *Bacillus anthracis* [26]. Here we present an extreme example of the canSNP concept where a small number of SNPs is used to distinguish between strains from a relatively small endemic region. For that purpose we replaced the 89 real-time PCR-based SNP typing assays by ten strategically placed canSNPs that enabled a differentiation of the ten described *M. ulcerans* haplotypes in our panel of strains.

The detected canSNPs provide useful genetic markers for the geographical and temporal analysis of *M. ulcerans* variants in the Densu River Valley of Ghana and the established TSP assays are now routinely used in the laboratories of the Noguchi Memorial Institute for *M. ulcerans* haplotype identification. However, a comprehensive overview of the *M. ulcerans* population structure in Africa can only be achieved by genome sequencing of *M. ulcerans* strains from other African BU endemic regions and subsequent identification of local sets of informative SNPs for further phylogenetic analyses.

## Supporting Information

Figure S1 **Setup of TSP SNP typing assays.** TSP endpoint detection by analysis of PCR product sizes on ethidium bromide-stained agarose gels for all ten TSP SNP typing assays. PCR products are shown for haplotypes 1–10 (lanes 1–10).
(TIF)

Figure S2 **TSP typing of clinical** ***M. ulcerans*** **isolates.** TSP endpoint detection by analysis of PCR product sizes on ethidium bromide-stained agarose gels for all ten TSP SNP typing assays. PCR products are shown for NM167, NM187, NM209, NM219, NM229, NM230, NM232, NM236, NM237, NM238(4), NM285, NM310, NM311, NM312, NM340, NM377, NM421C, NM465, NM491C, NM555, NM561, NM579, NM585, NAS or LS amplification control (lanes 1–24).
(TIF)

Table S1 **Temperature switch PCR locus-specific (LS) and nested allele specific (NAS) primers.** Overview of SNP loci (0 = reference allele, 1 = SNP allele), TSP-PCR primer IDs, sequences and melting temperatures as well as PCR product sizes for each of the ten TSP assays.
(DOC)

## References

[1] Johnson PDR, Stinear T, Small PLC, Pluschke G, Merritt RW, et al (2005) Buruli Ulcer (M. ulcerans Infection): New Insights, New Hope for Disease Control. PLoS Med 2: e108 doi:10.1371/journal.pmed.0020108 15839744 10.1371/journal.pmed.0020108 PMC1087202

[2] Stinear TP, Hong H, Frigui W, Pryor MJ, Brosch R, et al (2005) Common evolutionary origin for the unstable virulence plasmid pMUM found in geographically diverse strains of Mycobacterium ulcerans. J Bacteriol 187: 1668–1676 doi:10.1128/JB.187.5.1668-1676.2005 15716437 10.1128/JB.187.5.1668-1676.2005 PMC1064021

[3] Yip MJ, Porter JL, Fyfe JAM, Lavender CJ, Portaels F, et al (2007) Evolution of Mycobacterium ulcerans and other mycolactone-producing mycobacteria from a common Mycobacterium marinum progenitor. J Bacteriol 189: 2021–2029 doi:10.1128/JB.01442-06 17172337 10.1128/JB.01442-06 PMC1855710

[4] Qi W, Käser M, Röltgen K, Yeboah-Manu D, Pluschke G (2009) Genomic Diversity and Evolution of Mycobacterium ulcerans Revealed by Next-Generation Sequencing. PLoS Pathog 5 doi:10.1371/journal.ppat.1000580 10.1371/journal.ppat.1000580 PMC2736377 19806175

[5] Hong H, Coutanceau E, Leclerc M, Caleechurn L, Leadlay PF, et al (2008) Mycolactone Diffuses from Mycobacterium ulcerans–Infected Tissues and Targets Mononuclear Cells in Peripheral Blood and Lymphoid Organs. PLoS Negl Trop Dis 2 doi:10.1371/journal.pntd.0000325 10.1371/journal.pntd.0000325 PMC2565835 18941518

[6] Fyfe JAM, Lavender CJ, Handasyde KA, Legione AR, O'Brien CR, et al (2010) A Major Role for Mammals in the Ecology of Mycobacterium ulcerans. PLoS Negl Trop Dis 4: e791 doi:10.1371/journal.pntd.0000791 20706592 10.1371/journal.pntd.0000791 PMC2919402

[7] Lavender CJ, Fyfe JAM, Azuolas J, Brown K, Evans RN, et al (2011) Risk of Buruli Ulcer and Detection of Mycobacterium ulcerans in Mosquitoes in Southeastern Australia. PLoS Negl Trop Dis 5 doi:10.1371/journal.pntd.0001305 10.1371/journal.pntd.0001305 PMC3176747 21949891

[8] Monot M, Honoré N, Garnier T, Zidane N, Sherafi D, et al (2009) Comparative genomic and phylogeographic analysis of Mycobacterium leprae. Nature Genetics 41: 1282–1289 doi:10.1038/ng.477 19881526 10.1038/ng.477

[9] Maharjan RP, Gu C, Reeves PR, Sintchenko V, Gilbert GL, et al (2008) Genome-wide analysis of single nucleotide polymorphisms in Bordetella pertussis using comparative genomic sequencing. Research in Microbiology 159: 602–608 doi:10.1016/j.resmic.2008.08.004 18790049 10.1016/j.resmic.2008.08.004

[10] Morelli G, Song Y, Mazzoni CJ, Eppinger M, Roumagnac P, et al (2010) Phylogenetic diversity and historical patterns of pandemic spread of Yersinia pestis. Nat Genet 42: 1140–1143 doi:10.1038/ng.705 21037571 10.1038/ng.705 PMC2999892

[11] Kuroda M, Serizawa M, Okutani A, Sekizuka T, Banno S, et al (2010) Genome-Wide Single Nucleotide Polymorphism Typing Method for Identification of Bacillus Anthracis Species and Strains Among B. Cereus Group Species. J Clin Microbiol 48: 2821–2829 doi:10.1128/JCM.00137-10 20554827 10.1128/JCM.00137-10 PMC2916593

[12] Hilty M, Yeboah-Manu D, Boakye D, Mensah-Quainoo E, Rondini S, et al (2006) Genetic diversity in Mycobacterium ulcerans isolates from Ghana revealed by a newly identified locus containing a variable number of tandem repeats. J Bacteriol 188: 1462–1465 doi:10.1128/JB.188.4.1462-1465.2006 16452429 10.1128/JB.188.4.1462-1465.2006 PMC1367230

[13] Röltgen K, Qi W, Ruf M-T, Mensah-Quainoo E, Pidot SJ, et al (2010) Single nucleotide polymorphism typing of Mycobacterium ulcerans reveals focal transmission of buruli ulcer in a highly endemic region of Ghana. PLoS Negl Trop Dis 4: e751 doi:10.1371/journal.pntd.0000751 20652033 10.1371/journal.pntd.0000751 PMC2907412

[14] Käser M, Ruf M-T, Hauser J, Pluschke G (2010) Optimized DNA preparation from mycobacteria. Cold Spring Harb Protoc 2010: pdb.prot5408 doi:10.1101/pdb.prot5408 20360362 10.1101/pdb.prot5408

[15] Hayden M, Tabone T, Mather D (2009) Development and assessment of simple PCR markers for SNP genotyping in barley. TAG Theoretical and Applied Genetics 119: 939–951 doi:10.1007/s00122-009-1101-7 19597725 10.1007/s00122-009-1101-7

[16] Tabone T, Mather DE, Hayden MJ (2009) Temperature switch PCR (TSP): Robust assay design for reliable amplification and genotyping of SNPs. BMC Genomics 10: 580 doi:10.1186/1471-2164-10-580 19958555 10.1186/1471-2164-10-580 PMC2795770

[17] Untergasser A, Nijveen H, Rao X, Bisseling T, Geurts R, et al (2007) Primer3Plus, an enhanced web interface to Primer3. Nucleic Acids Res 35: W71–74 doi:10.1093/nar/gkm306 17485472 10.1093/nar/gkm306 PMC1933133

[18] Käser M, Rondini S, Naegeli M, Stinear T, Portaels F, et al (2007) Evolution of two distinct phylogenetic lineages of the emerging human pathogen Mycobacterium ulcerans. BMC Evolutionary Biology 7: 177 doi:10.1186/1471-2148-7-177 17900363 10.1186/1471-2148-7-177 PMC2098775

[19] Rondini S, Käser M, Stinear T, Tessier M, Mangold C, et al (2007) Ongoing genome reduction in Mycobacterium ulcerans. Emerging Infect Dis 13: 1008–1015. 18214172 10.3201/eid1307.060205 PMC2878211

[20] Stinear T, Davies JK, Jenkin GA, Portaels F, Ross BC, et al (2000) A simple PCR method for rapid genotype analysis of Mycobacterium ulcerans. J Clin Microbiol 38: 1482–1487. 10747130 10.1128/jcm.38.4.1482-1487.2000 PMC86470

[21] Chemlal K, De Ridder K, Fonteyne PA, Meyers WM, Swings J, et al (2001) The use of IS2404 restriction fragment length polymorphisms suggests the diversity of Mycobacterium ulcerans from different geographical areas. Am J Trop Med Hyg 64: 270–273. 11463115 10.4269/ajtmh.2001.64.270

[22] Ablordey A, Kotlowski R, Swings J, Portaels F (2005) PCR amplification with primers based on IS2404 and GC-rich repeated sequence reveals polymorphism in Mycobacterium ulcerans. J Clin Microbiol 43: 448–451 doi:10.1128/JCM.43.1.448-451.2005 15635012 10.1128/JCM.43.1.448-451.2005 PMC540136

[23] Stragier P, Ablordey A, Meyers WM, Portaels F (2005) Genotyping Mycobacterium ulcerans and Mycobacterium marinum by using mycobacterial interspersed repetitive units. J Bacteriol 187: 1639–1647 doi:10.1128/JB.187.5.1639-1647.2005 15716434 10.1128/JB.187.5.1639-1647.2005 PMC1064023

[24] Ablordey A, Fonteyne P-A, Stragier P, Vandamme P, Portaels F (2007) Identification of a new variable number tandem repeat locus in Mycobacterium ulcerans for potential strain discrimination among African isolates. Clin Microbiol Infect 13: 734–736 doi:10.1111/j.1469-0691.2007.01716.x 17403131 10.1111/j.1469-0691.2007.01716.x

[25] Käser M, Gutmann O, Hauser J, Stinear T, Cole S, et al (2009) Lack of insertional-deletional polymorphism in a collection of Mycobacterium ulcerans isolates from Ghanaian Buruli ulcer patients. J Clin Microbiol 47: 3640–3646 doi:10.1128/JCM.00760-09 19726605 10.1128/JCM.00760-09 PMC2772640

[26] Keim P, Van Ert MN, Pearson T, Vogler AJ, Huynh LY, et al (2004) Anthrax molecular epidemiology and forensics: using the appropriate marker for different evolutionary scales. Infect Genet Evol 4: 205–213 doi:10.1016/j.meegid.2004.02.005 15450200 10.1016/j.meegid.2004.02.005

[27] Van Ert MN, Easterday WR, Huynh LY, Okinaka RT, Hugh-Jones ME, et al (2007) Global genetic population structure of Bacillus anthracis. PLoS ONE 2: e461 doi:10.1371/journal.pone.0000461 17520020 10.1371/journal.pone.0000461 PMC1866244

[28] Vogler AJ, Birdsell D, Price LB, Bowers JR, Beckstrom-Sternberg SM, et al (2009) Phylogeography of Francisella tularensis: global expansion of a highly fit clone. J Bacteriol 191: 2474–2484 doi:10.1128/JB.01786-08 19251856 10.1128/JB.01786-08 PMC2668398

[29] van Gent M, Bart MJ, van der Heide HGJ, Heuvelman KJ, Kallonen T, et al (2011) SNP-Based Typing: A Useful Tool to Study Bordetella pertussis Populations. PLoS ONE 6: e20340 doi:10.1371/journal.pone.0020340 21647370 10.1371/journal.pone.0020340 PMC3103551

[30] Vogler AJ, Chan F, Wagner DM, Roumagnac P, Lee J, et al (2011) Phylogeography and molecular epidemiology of Yersinia pestis in Madagascar. PLoS Negl Trop Dis 5: e1319 doi:10.1371/journal.pntd.0001319 21931876 10.1371/journal.pntd.0001319 PMC3172189

[31] Easterday WR, Van Ert MN, Zanecki S, Keim P (2005) Specific detection of bacillus anthracis using a TaqMan mismatch amplification mutation assay. Bio Techniques 38: 731–735. 10.2144/05385ST03 15945372

[32] Van Ert MN, Easterday WR, Simonson TS, U'Ren JM, Pearson T, et al (2007) Strain-specific single-nucleotide polymorphism assays for the Bacillus anthracis Ames strain. J Clin Microbiol 45: 47–53 doi:10.1128/JCM.01233-06 17093023 10.1128/JCM.01233-06 PMC1828967

[33] Vogler AJ, Driebe EM, Lee J, Auerbach RK, Allender CJ, et al (2008) Assays for the rapid and specific identification of North American Yersinia pestis and the common laboratory strain CO92. Bio Techniques 44: 201, 203–204, 207. 10.2144/000112815 PMC3836605 18330347

