# Supplementary figures and images for: Development of a Temperature-Switch PCR-Based SNP Typing Method for Mycobacterium ulcerans
Source: PLoS Negl Trop Dis. 2012 Nov 15;6(11):e1904. doi: 10.1371/journal.pntd.0001904 (PMC3499370; doi:10.1371/journal.pntd.0001904)

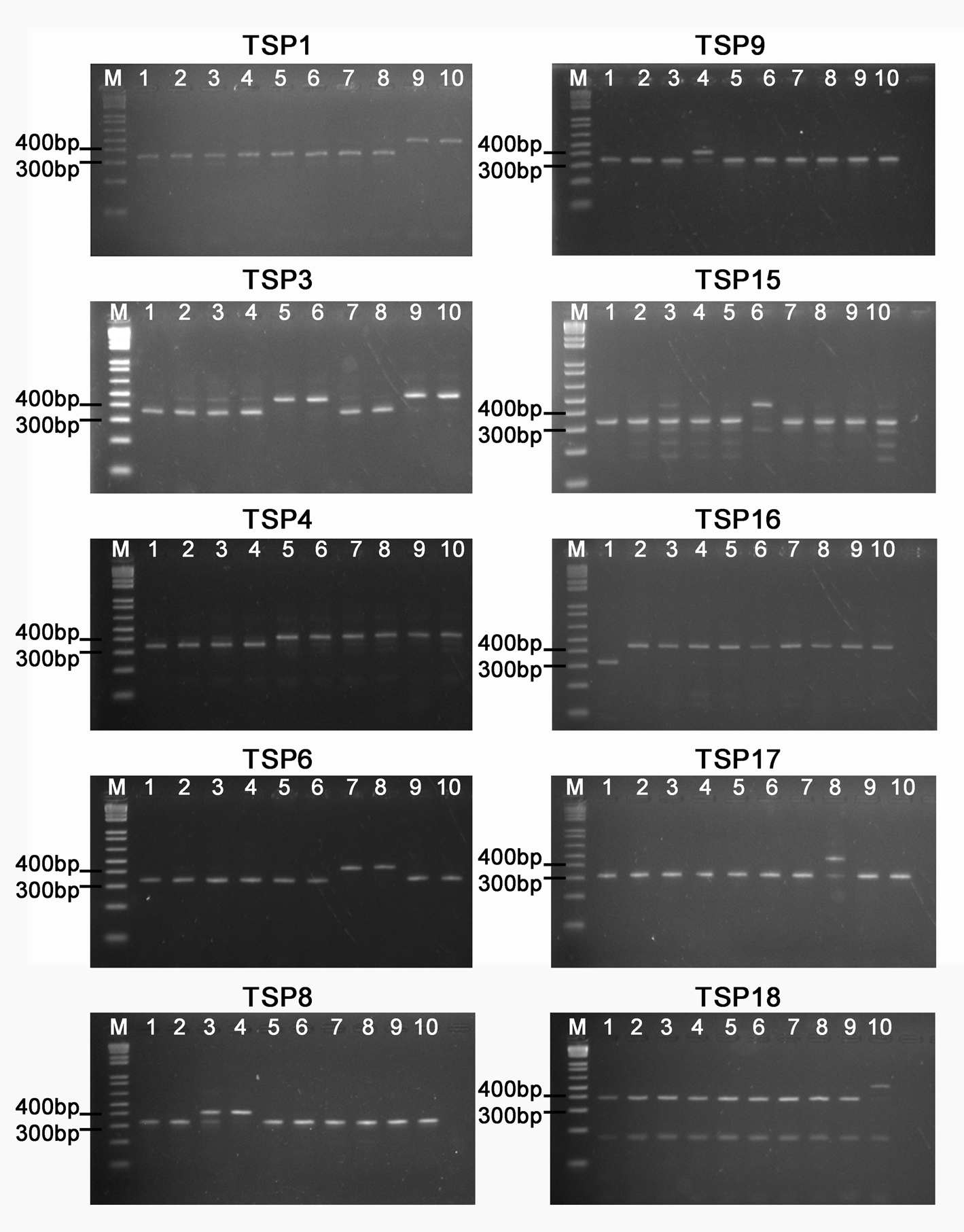

Supplement: Figure S1 — Setup of TSP SNP typing assays. TSP endpoint detection by analysis of PCR product sizes on ethidium bromide-stained agarose gels for all ten TSP SNP typing assays. PCR products are shown for haplotypes 1–10 (lanes 1–10). (TIF) [file pntd.0001904.s001.tif]

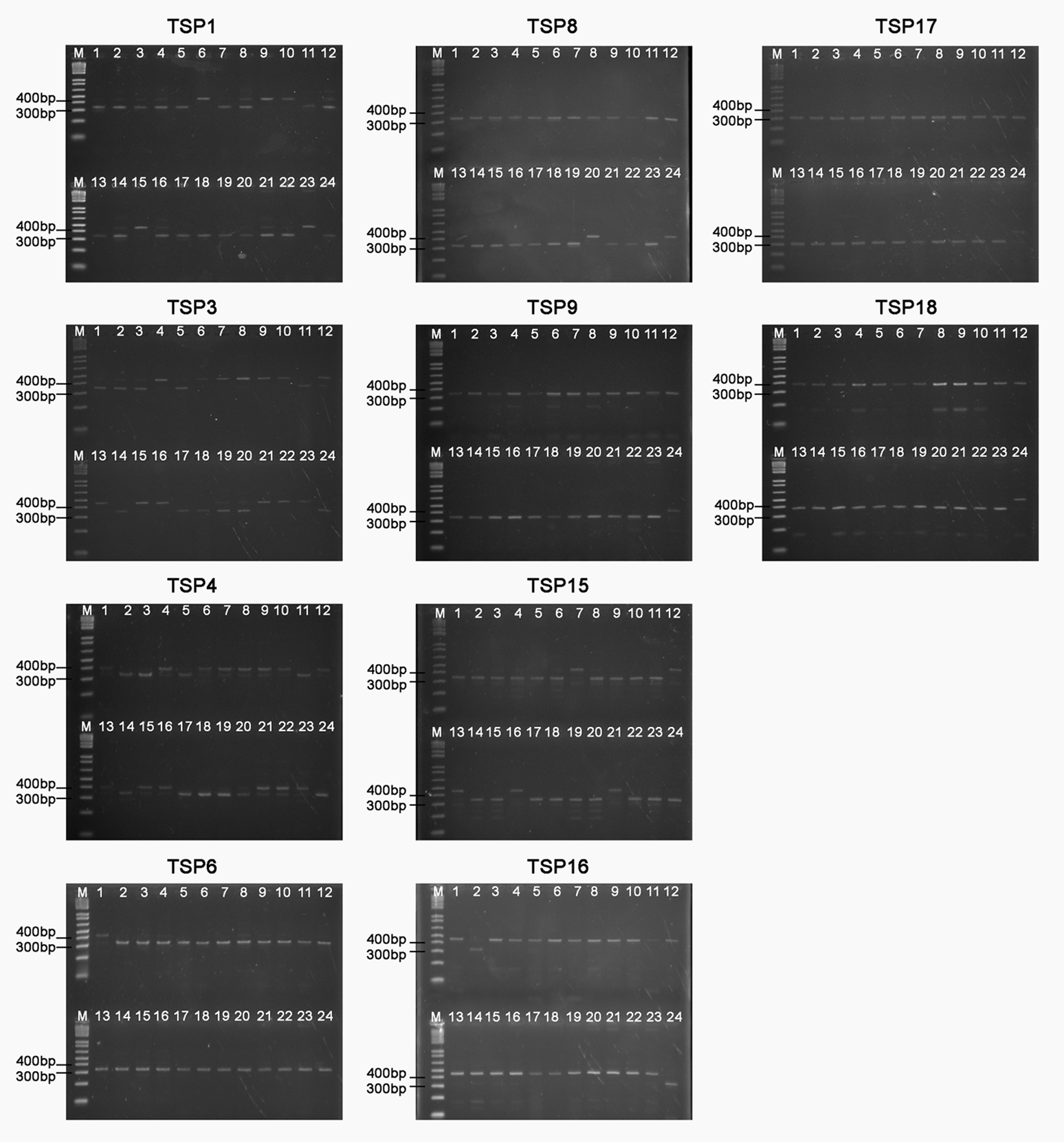

Supplement: Figure S2 — TSP typing of clinical M. ulcerans isolates. TSP endpoint detection by analysis of PCR product sizes on ethidium bromide-stained agarose gels for all ten TSP SNP typing assays. PCR products are shown for NM167, NM187, NM209, NM219, NM229, NM230, NM232, NM236, NM237, NM238(4), NM285, NM310, NM311, NM312, NM340, NM377, NM421C, NM465, NM491C, NM555, NM561, NM579, NM585, NAS or LS amplification control (lanes 1–24). (TIF) [file pntd.0001904.s002.tif]
