# Supplementary material for: Development of a Temperature-Switch PCR-Based SNP Typing Method for Mycobacterium ulcerans
Source: PLoS Negl Trop Dis. 2012 Nov 15;6(11):e1904. doi: 10.1371/journal.pntd.0001904 (PMC3499370; doi:10.1371/journal.pntd.0001904)
Supplement: Table S1 — Temperature switch PCR locus-specific (LS) and nested allele specific (NAS) primers. Overview of SNP loci (0 = reference allele, 1 = SNP allele), TSP-PCR primer IDs, sequences and melting temperatures as well as PCR product sizes for each of the ten TSP assays. (DOC) [file pntd.0001904.s003.doc]

**Table S1. Temperature switch PCR locus-specific (LS) and nested allele specific (NAS) primers.**

| **Genome position 0/1** | **Primer ID** | **Primer sequence** | **Melting temperature (°C)** | **LS-LS and NAS-LS PCR product size (bp)** |
| --- | --- | --- | --- | --- |
|  |  |  |  |  |
| **5603344 A/C** | LS forward 1 | ggcgcagatttccgatctt | 62.54 | 459 |
|  | NAS forward 1 | tccgagaatccgatca | 45.14🡪53.82 | 339 |
|  | LS reverse 1 | gcgttaatcgtcacggatgc | 63.77 |  |
| **2455125 T/C** | LS forward 3 | caacagggcaccttcagctc | 63.27 | 457 |
|  | NAS forward 3 | tggctcccacaggtt | 43.18🡪53.57 | 353 |
|  | LS reverse 3 | ctggtcttgtcgtgcagcag | 63.21 |  |
| **2827516 T/C** | LS forward 4 | gtaagggctctcgccactca | 62.79 | 401 |
|  | NAS forward 4 | tgggatgcgtcatttt | 44.29🡪53.82 | 339 |
|  | LS reverse 4 | gctggcgacgatgagcag | 64.38 |  |
| **5558163 A/C** | LS forward 6 | gacgcactggacctgattcc | 63.02 | 427 |
|  | NAS forward 6 | tgtcggcctttacga | 46.42🡪52.25 | 333 |
|  | LS reverse 6 | aaggtcccgtggcagtgtt | 62.89 |  |
| **749081 T/C** | LS forward 8 | ggcgtaggaggcgaacagt | 62.68 | 415 |
|  | NAS forward 8 | caagtggcccagatgt | 46.37🡪53.12 | 330 |
|  | LS reverse 8 | accgatctacttgccgatgg | 62.32 |  |
| **2041755 G/A** | LS forward 9 | atgcccgggagtcttgga | 63.97 | 426 |
|  | NAS forward 9 | tggtatgtagagggtcagg | 44.6🡪53.28 | 346 |
|  | LS reverse 9 | aaccacctgcaatcccacac | 63.15 |  |
| **4028025 T/C** | LS forward 15 | GGTCCTCGGGCTTCTCGT | 62.73 | 457 |
|  | NAS forward 15 | tgTGGCCTACCGGAT | 47.89🡪53.58 | 341 |
|  | LS reverse 15 | GCAGCACGAAAGCCAACC | 62.94 |  |
| **453370 A/G** | LS forward 16 | gaacagatgccggtgcagat | 63.53 | 452 |
|  | NAS forward 16 | ggtttcggcgaagtaa | 46.12🡪52.83 | 326 |
|  | LS reverse 16 | ggctcggtgagatgtgtgg | 62.76 |  |
| **3993860 A/G** | LS forward 17 | cgcgtcgaactcgaagaagt | 62.93 |  |
|  | NAS reverse 17 | cactcgaagtgacggtt | 46.86🡪52.46 | 314 |
|  | LS reverse 17 | ctgggcgtcaaaggttttca | 63.36 | 464 |
| **2056416 G/A** | LS forward 18 | cgctctcgaggttttgcagt | 62.93 |  |
|  | NAS reverse 18 | ttaccgcaacggctc | 52.28🡪54.83 | 387 |
|  | LS reverse 18 | tgactgatggtgccggtgt | 63.71 | 523 |
